# Cardiogenic shock with highly complicated course after influenza A virus infection treated with vva-ECMO and Impella CP (ECMELLA): a case report

**DOI:** 10.1186/s12872-021-02346-2

**Published:** 2021-11-08

**Authors:** Daniel Ebert, Nils Mungard, Alexander Mensch, Lorenz Homeister, Jan Willsch, Richard Ibe, Henning Baust, Markus Stiller, Artur Rebelo, Joerg Ukkat, Angelos G. Rigopoulos, Elke Weber, Michael Bucher, Michel Noutsias

**Affiliations:** 1 Department of Anesthesiology, University Hospital Halle, Martin-Luther-University Halle-Wittenberg, Ernst-Grube-Strasse 40, 06120 Halle (Saale), Germany; 2 Department of Neurology, University Hospital Halle, Martin-Luther-University Halle-Wittenberg, Ernst-Grube-Strasse 40, 06120 Halle (Saale), Germany; 3 Department of Cardiac Surgery, Mid-German Heart Center, University Hospital Halle, Martin-Luther-University Halle-Wittenberg, Ernst-Grube-Strasse 40, 06120 Halle (Saale), Germany; 4 Department of Vascular Surgery, University Hospital Halle, Martin-Luther-University Halle-Wittenberg, Ernst-Grube-Strasse 40, 06120 Halle (Saale), Germany; 5 Division of Cardiology, Angiology and Intensive Medical Care, Department of Internal Medicine III (KIM III), Mid-German Heart Center, Faculty of Medicine, University Hospital Halle, Martin-Luther-University Halle-Wittenberg, Ernst-Grube-Strasse 40, 06120 Halle (Saale), Germany; 6 Department of Adult Cardiology, Mitera General Hospital, Hygeia Group, 6 Erythrou Stavrou Street, 15123 Marousi, Athens Greece; 7 Department of Anesthesiology, St. Elisabeth & St. Barbara Hospital Halle, Mauerstrasse 5, 06110 Halle (Saale), Germany

**Keywords:** Cardiogenic shock, Case report, ECMO, Impella, Mechanical circulatory support, Myocarditis, Recovery

## Abstract

**Background:**

The value of mechanical circulatory support (MCS) in cardiogenic shock, especially the combination of the ECMELLA approach (Impella combined with ECMO), remains controversial.

**Case presentation:**

A previously healthy 33-year-old female patient was submitted to a local emergency department with a flu-like infection and febrile temperatures up to 39 °C. The patient was tested positive for type-A influenza, however negative for SARS-CoV-2. Despite escalated invasive ventilation, refractory hypercapnia (paCO_2_: 22 kPa) with severe respiratory acidosis (pH: 6.9) and a rising norepinephrine rate occurred within a few hours. Due to a Horovitz-Index < 100, out-of-centre veno-venous extracorporeal membrane oxygenation (vv-ECMO)-implantation was performed. A CT-scan done because of anisocoria revealed an extended dissection of the right vertebral artery. While the initial left ventricular function was normal, echocardiography revealed severe global hypokinesia. After angiographic exclusion of coronary artery stenoses, we geared up LV unloading by additional implantation of an Impella CP and expanded the vv-ECMO to a veno-venous-arterial ECMO (vva-ECMO). Clinically relevant bleeding from the punctured femoral arteries resulted in massive transfusion and was treated by vascular surgery later on. Under continued MCS, LVEF increased to approximately 40% 2 days after the initiation of ECMELLA. After weaning, the Impella CP was explanted at day 5 and the vva-ECMO was removed on day 9, respectively. The patient was discharged in an unaffected neurological condition to rehabilitation 25 days after the initial admission.

**Conclusions:**

This exceptional case exemplifies the importance of aggressive MCS in severe cardiogenic shock, which may be especially promising in younger patients with non-ischaemic cardiomyopathy and potentially reversible causes of cardiogenic shock. This case impressively demonstrates that especially young patients may achieve complete neurological restoration, even though the initial prognosis may appear unfavourable.

## Background

Mechanical circulatory support (MCS) may contribute to improve the grave prognosis of cardiogenic shock. The avenue of combination of several systems such as extracorporeal membrane oxygenation (ECMO) with the Impella (ECMELLA) gave rise to complex MCS approaches which might contribute the reduced mortality despite higher complication rates [1]. However, defining the patient profiles who might likely profit most from such MCS-regimens, favourable timing of MCS interventions, including further important issues such as the initially unclear neurological outcome, remain intriguing tasks for the complex decision process [2].

## Case presentation

**Fig. 1:**
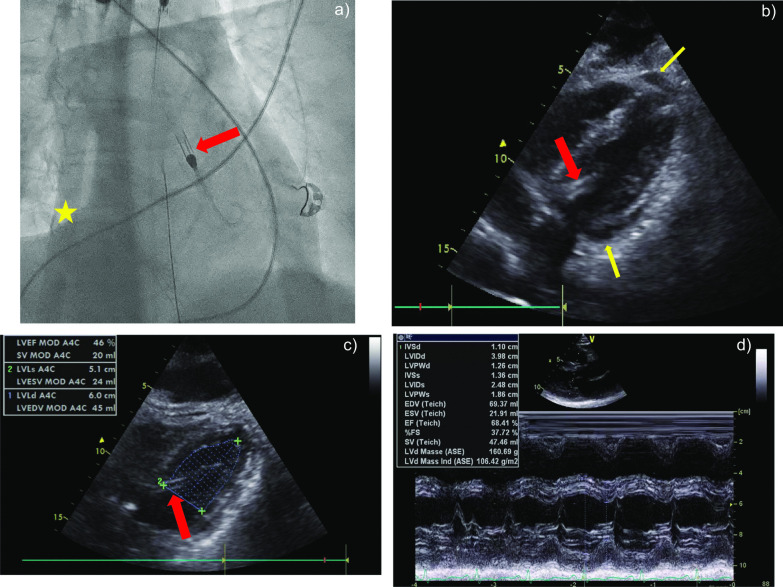
Aspects of ECMELLA treatment. **a** Fluoroscopy of the Impella CP implanted in the left ventricle (red arrow), and the venous cannula of the ECMO implanted in the inferior vena cava and in the right atrium (yellow asterisk). **b** TTE showing the severely hypokinetic left ventricle with the implanted Impella CP (red arrow) immediately after completion of the ECMELLA concept. Please note the non-compressing circular pericardial effusion (PE; yellow arrows). **c** TTE showing the risen LVEF (ca. 40%) 2 days after the initiation of ECMELLA. Please note the Impella CP in the LV (red arrow). **d** 2D echocardiography showing recovery of LVEF (68%) and LV dimensions after severe cardiogenic shock treated with ECMELLA. No residual PE is discernible

**Fig. 2:**
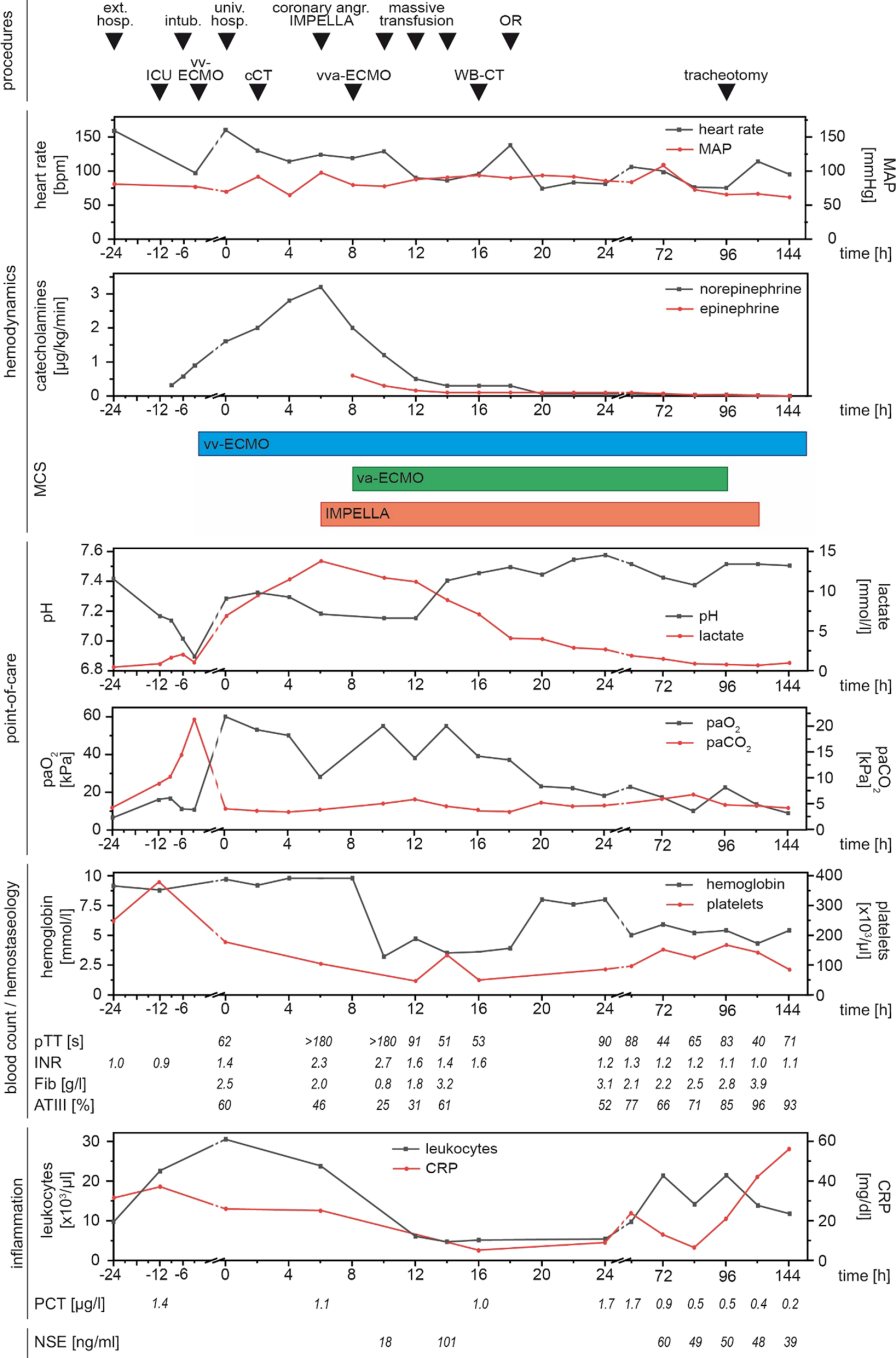
Time course of cardiogenic shock, ECMELLA and ICU treatment. Chronological events and applied procedures in context of the underlying hemodynamics, point-of-care and laboratory parameters. The time period from referral to external hospital up to 144 h after admission to university hospital is shown. Note the remarkable respiratory acidosis with excessive elevated paCO_2_ before vv-ECMO implantation, the significant reduction of catecholamines under ECMELLA therapy and the impressive derangement in haemostatic parameters related to bleeding complications demanding massive transfusion. Due to a better overview, 1 cCT and 1 WB-CT, immediately performed after vv-ECMO implantation, are not shown in the figure. *ext. hosp., univ. hosp.* Admission to external/university hospital, *ICU* intensive care unit, *intub.* intubation, *coronary angr.* coronary angiography, *vv(a)-ECMO* veno-venous (arterial) extracorporeal membrane oxygenation, *cCT* cranial CT-scan, *WB-CT* whole body CT-scan, *OR* operation room, *MAP* mean arterial pressure, *MCS* mechanical circulatory support, *pTT* partial thromboplastin time, *INR* international normalized ratio, *Fib* fibrinogen, *ATIII* antihrombin III, *CRP* C-reactive protein, *PCT* procalcitonin, *NSE* neuron specific enolase

A previously healthy 33-year-old female patient was submitted to a local emergency department with a flu-like infection and febrile temperatures up to 39 °C. The medical history solely contained a pre-existing bronchial asthma. The nasal swab tested negative for SARS-CoV-2 and positive for type-A influenza. Further 2 swabs of bronchoalveolar lavage were negative for SARS-CoV-2. Treatment with oseltamivir was initiated 1 h after receiving the laboratory report for type-A influenza. Just a few hours later, her respiratory insufficiency worsened, and the patient was admitted to the intensive care unit. After further respiratory deterioration under non-invasive ventilation, endotracheal intubation was performed. Despite escalated invasive ventilation, refractory hypercapnia (paCO_2_: 22 kPa) with severe respiratory acidosis (pH: 6.9) occurred within a few hours. A CT scan of the lungs showed swollen bronchioles, however, acute respiratory distress syndrome (ARDS) like infiltrates were absent. Reversible causes like tension pneumothorax, cardiac tamponade or pulmonary embolism were ruled out. Because of a transitory anisocoria the radiological diagnostics were supplemented with a native CT-scan of the brain, showing a mild cerebral oedema. In correspondence with a highly reduced pulmonary compliance, persisting hypercapnia and a Horovitz-Index < 100, out-of-centre veno-venous-ECMO (vv-ECMO)-implantation was performed due to a presumed refractory status asthmaticus. Immediately after ECMO-implantation, multiple episodes of tachycardia due to atrial fibrillation (Afib) had to be terminated by electrical cardioversion and amiodarone. However, the Afib episodes contributed to progressive haemodynamic instability. The transoesophageal echocardiography (TOE) initially showed hyperdynamic ventricular contractility with preserved left ventricular ejection fraction (LVEF), compatible with a sepsis-like constellation. Though hypercapnia was rapidly declining during ECMO-therapy, anisocoria and hypotension continued. This prompted a further whole-body CT-scan, which ruled out aggravation of cerebral oedema or any abdominal or thoracic bleeding after ECMO-cannulation as possible causes. The patient was transferred to our university hospital with a high norepinephrine rate (1.6 mcg/kg/min) for further complex intensive care treatment. We initiated an empiric treatment with meropenem and clarithromycin in accordance with a presumed septic shock following concomitant community-acquired pneumonia. Aiming at diagnosing the origin of persisting anisocoria, a third CT-scan of the brain with contrast medium was carried out, which now revealed an extended dissection of the right vertebral artery. Somatosensory evoked potentials (SEP) were conducted, showing an extremely reduced cortical action potential (N20) in agreement with a distinct brainstem damage. Due to rising doses of norepinephrine (Fig. 2), we carried out transthoracic echocardiography (TTE), which revealed severe global hypokinesia and LVEF 19%, consistent with severe cardiogenic shock. There was no hint for regional hypokinesia patterns consistent with takotsubo syndrome (TTS). After angiographic exclusion of relevant coronary artery stenoses, we decided to support left ventricular function and LV unloading by gearing up MCS [1], and implanted an Impella CP in the heart catheterization laboratory. The patient required epinephrine in addition to rising doses of norepinephrine (Fig. 2), which prompted us to subsequently expand to veno-venous-arterial ECMO (vva-ECMO) in terms of a vva-ECMELLA (Fig. 1a). The TTE performed immediately after completion of ECMELLA revealed a severely depressed LVEF of approximately 15%, confirmed the right position of Impella CP in the LV and identified circular pericardial effusion without evidence for compression of the right ventricle or right atrium (no sign of pericardial tamponade; Fig. 1b). Under ECMELLA, the haemodynamic situation of the patient improved on the ICU, accompanied by substantially decreasing catecholamines infusion rates. The complex clinical course of the patient is illustrated in Fig. 2. Coagulation was severely compromised related to high-dose heparin administration for the pertinent anticoagulation of the ECMELLA and the incidental hypothermia, contributing to spontaneous bleeding from mucous membranes and all punctured lines, demanding a massive transfusion. Unfortunately, bleeding from the punctured sites of the bilateral femoral arteries aggravated, so that the patient was subjected to vascular surgical treatment, which substantially improved the bleeding situation. From out-of-hospital ECMO-implantation and up until surgical bleeding control, the patient received 19 units of packed red blood cells, 17 units of fresh frozen plasma, 3 units of high concentrated platelets and multiple coagulation factors (Fig. 2). The applied catecholamines could be reduced substantially in the subsequent days under ECMELLA. Coincidentally, a severe orbital compartment syndrome due to an initially anticoagulation-related retrobulbar hematoma resulted in a central retinal artery occlusion. Despite lateral canthotomy and orbital decompression, a right-sided amaurosis unfortunately remained in the course. Further on, MCS led to a step-by-step cardiac recompensation with increasing LVEF (to approximately 40%) in TTE 2 days after the initiation of ECMELLA (Fig. 1c). Aiming first to reduce the left ventricular afterload, the arterial cannula of the vva-ECMO was explanted after weaning of the ECMO on day 4, followed by removal of the Impella CP one day later after uncomplicated weaning from “auto-mode” with 3.5 l/min to P2. To facilitate respiratory weaning, dilatative tracheotomy was performed and subsequently vv-ECMO weaning was carried out without any complications on day 9. After total removal of MCS, echocardiography showed a completely restored LV function (Fig. 1d). No neurological impairments were present after reduction of sedation. Along this line, under supportive intensive care treatment the patient was decannulated 12 days after tracheotomy and was transferred to the peripheral cardiology ward. Her NT-proBNP, which had peaked at 4791 pg/ml 3 days after referral to our hospital, had decreased to 601 pg/ml 5 days before being discharged to rehabilitation (25 days after the initial admission to hospital) in a neurologically unaffected condition. The 33-year-old female patient was very grateful for having survived the severe, life-threatening cardiogenic shock and being able to meet her children (1 and 3 years old) and her husband again.

## Discussion and conclusions

This exceptional case of a fairly young woman without prior multimorbidity exemplifies the importance of aggressive MCS in severe cardiogenic shock, which may be especially promising in younger patients with non-ischaemic cardiomyopathy and potentially reversible cause of cardiogenic shock (e.g. viral myocarditis), who may develop maximal plasticity of LV recovery [3, 4]. The patient might have had myocarditis associated with influenza A virus, which might explain the new onset of highly depressed LVEF in terms of a fulminant myocarditis [5], as well as the initial pericardial effusion (PE), which resolved spontaneously during the dynamic natural course of the disease [5]. Aspects of septic cardiomyopathy, which are not mutually exclusive, might also have contributed to the acute condition, as well as to the recovery course of the patient [6]. Since the ECMO and Impella are not compatible with cardiac magnetic resonance (CMR), we did not have the opportunity to perform CMR in this particular case to ascertain the differential diagnosis of myocarditis. In a major proportion of patients with clinically suspected myocarditis, symptomatic heart failure treatment without additional immunomodulatory treatment is the mainstay of evidence-based medicine, while endomyocardial biopsy (EMB) and immunosuppressive treatment may be eligible in rare cases not responding to maximum heart failure treatment, including MCS, which may be compatible with giant-cell myocarditis [5, 7, 8]. Considering the substantial clinical improvement under ECMELLA, we can largely exclude giant-cell myocarditis in this patient, since this condition would at least need immunosuppressive treatment to improve, which was applicable in this case [8, 9]. Therefore, omitting EMB diagnostics, which may also impose periinterventional complications [10], is appropriate in this patient [7, 8], and the implementation of MCS may be deemed more important in rapidly deteriorating cardiogenic shock. TTS is a further clinical scenario which needs to be considered as differential diagnosis of cardiogenic shock following physical triggers [11]. Noticeably, TTS patients presenting with cardiogenic shock have substantially impaired prognosis [12]. However, there was no hint for regional hypokinesia patterns assessed by echocardiography consistent with TTS in this patient. A further aspect of the clinical presentation of the patient might be of importance: Afib is associated with significantly increased risk of in-hospital mortality and complications including cardiogenic shock in patients with acute myocarditis [13]. Afib may be an issue with prognostic implications in patients with cardiogenic shock. In a recent analysis in consecutive patients with cardiogenic shock complicating acute myocardial infarction, patients with Afib already on admission showed higher all-cause mortality at 30 days and 1 year compared with patients with newly detected Afib during hospital stay [14]. However, the sole criterion of Afib may not be of overall prognostic importance in cardiogenic shock [15]. On the other hand, the potential role of va-ECMO for recovery from refractory cardiogenic shock in arrhythmia-induced non-ischemic cardiomyopathy has been recently shown in 35 patients over 14 years [16]. In summary, several, not mutually exclusive aspects of the clinical scenario may have contributed to the complex pathogenic pathways possibly initiated by the myocarditis due to influenza A infection of this 33-year-old female patient and may also have aggravated her clinical course to refractory cardiogenic shock.

Periprocedural bleeding after percutaneous interventions using large-bore catheters such as implantation of peripheral percutaneous left ventricular assist device (pVAD; among others Impella, ECMO) is associated with increased mortality, blood transfusions, length of Stay and health care costs [17]. The incidence of hematoma, need for blood transfusions and reintervention was 10.1%, 17% and 2.6% in n = 1.816 patients treated by pVAD in this national database, respectively. pVAD patients with bleeding complications had a significantly higher mortality (35.4%) compared with pVAD patients without bleeding complications (29.6%). Of note, the mean age of the patients in this national database was 75.6 years. The substantially younger age of our 33-year-old patient may be also an important factor contributing to higher chances to survive complications being associated with pVAD implantation. One approach to address this pivotal issue may be the early detection of blood loss. Recently, an early bird bleed monitoring system has shown promising results in detection of intra- and postprocedural bleeding complications in endovascular procedures, showing also a high level of agreement with computed tomography scan [18].

Despite the possibility of substantial periinterventional complications, which may also require massive blood transfusion and surgical treatment, this case adds to our knowledge on the potentially decisive role of aggressive MCS, including ECMELLA, in rare patients with cardiogenic shock due to clinically suspected myocarditis. Myocarditis and pericarditis have been associated in ca. 14% and 19% of COVID-19 disease, respectively [19]. However, the negative PCR from 2 pharyngeal swabs and from 2 bronchoalveolar lavage largely rule out COVID-19 disease in this young patient treated during the first wave of COVID-19 outbreak (March 2020) in Germany. The precise role of MCS in these patients, aiding to overcome the critical phase of severe cardiogenic shock, enabling the transition to cardiac recompensation secondary to recovery from potentially reversible causes of cardiogenic shock (e.g. fulminant myocarditis, septic cardiomyopathy, TTS), warrants further evaluation [1–3, 12].

Concerning the initial neurological status, it was uncertain, how to classify the described clinical findings in the context of the extended dissection of the vertebral artery and a presumably hypoxic brain damage. Based on recommendations related to prognostic biomarkers, the neurological prognosis in our patient was expected to be rather poor, especially due to distinctly elevated and increasing NSE, a generalized cerebral oedema and latency-delayed, reduced cortical action potentials during conduction of evoked potentials. Therefore, it may be critical to withhold or withdraw maximum life-supporting therapies in the early stage of assumed hypoxemic brain damage especially in young adults because of data primarily derived from older patients [20]. At 6-month-follow-up our patient did not present any neurological impairments except for dysesthesia in both femoral regions. Interestingly, the stenosis of the vertebral artery dissection was no longer detectable by ultrasound and the initial diminished SEPs showed normalized latencies of cortical responses with increasing amplitudes.

Aggressive MCS, including vva-ECMELLA, may have a decisive role in survival after cardiogenic shock, and may successfully bridge to improvement of LVEF and to complete neurological restoration especially in young patients, despite potential periinterventional substantial complications and even though the initial prognosis may appear unfavourable. A broader, standardized implementation of MCS programs including ECMO and Impella following newly developed recommendations is warranted to meet the need of early MCS treatment in cardiogenic shock and the rapidly growing numbers of refractory respiratory failure especially during the current COVID-19 pandemic [2, 21, 22].

## Data Availability

Not applicable.

## Abbreviations

Afib: Atrial fibrillation
ARDS: Acute respiratory distress syndrome
CMR: Cardiac magnetic resonance
CT: Computed tomography
ECMELLA: ECMO combined with an Impella
ECMO: Extracorporeal membrane oxygenation
EMB: Endomyocardial biopsy
ICU: Intensive care unit
LVEF: Left ventricular ejection fraction
MCS: Mechanical circulatory support
NSE: Neuron-specific enolase
SEP: Somatosensory evoked potential
PE: Pericardial effusion
pVAD: Percutaneous left ventricular assist device
TOE: Transoesophageal echocardiography
TTE: Transthoracic echocardiography
TTS: Takotsubo syndrome
vv-ECMO: Veno-venous-ECMO
va-ECMO: Veno-arterial-ECMO
vva-ECMO: Veno-venous-arterial-ECMO
